# Directed Evolution of Stabilized Monomeric CD19 for Monovalent CAR Interaction Studies and Monitoring of CAR-T Cell Patients

**DOI:** 10.1021/acssynbio.1c00010

**Published:** 2021-04-12

**Authors:** Elisabeth Laurent, Anna Sieber, Benjamin Salzer, Anna Wachernig, Jacqueline Seigner, Manfred Lehner, René Geyeregger, Bernhard Kratzer, Ulrich Jäger, Renate Kunert, Winfried F. Pickl, Michael W. Traxlmayr

**Affiliations:** † Department of Biotechnology and BOKU Core Facility Biomolecular and Cellular Analysis, BOKU - University of Natural Resources and Life Sciences, Muthgasse 18, 1190 Vienna, Austria; ‡ Department of Biotechnology, BOKU - University of Natural Resources and Life Sciences, Muthgasse 18, 1190 Vienna, Austria; § St. Anna Children’s Cancer Research Institute, Zimmermannplatz 10, 1090 Vienna, Austria; ∥ Department of Chemistry, BOKU - University of Natural Resources and Life Sciences, Muthgasse 18, 1190 Vienna, Austria; ⊥ Institute of Immunology, Center for Pathophysiology, Infectiology and Immunology, Medical University of Vienna, Lazarettgasse 19, 1090 Vienna, Austria; # Department of Internal Medicine, Division of Hematology and Hemostaseology, Medical University of Vienna, Währinger Gürtel 18-20, 1090 Vienna, Austria

**Keywords:** CD19, chimeric antigen receptor (CAR), yeast surface display, directed evolution, protein engineering, FMC63

## Abstract

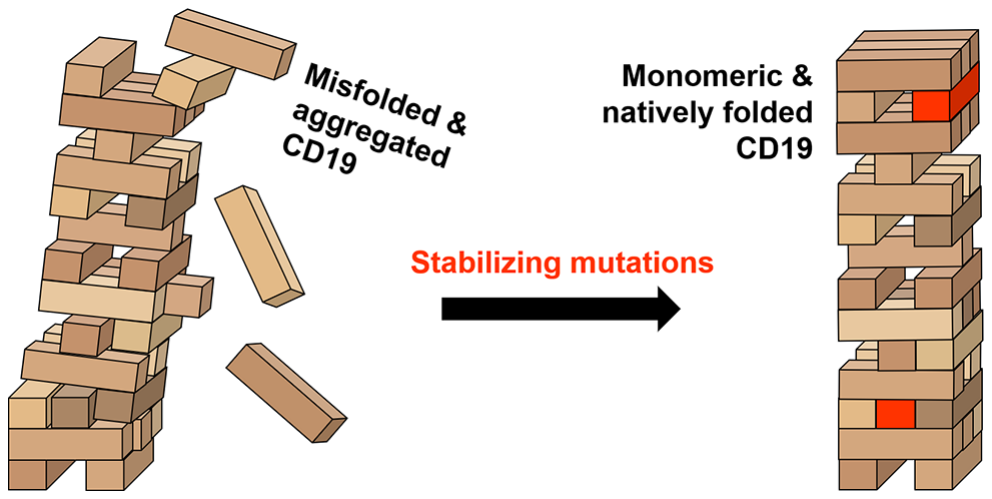

CD19 is among the most relevant targets in cancer immunotherapy. However, its extracellular domain (ECD) is prone to aggregation and misfolding, representing a major obstacle for the development and analysis of CD19-targeted therapeutics. Here, we engineered stabilized CD19-ECD (termed SuperFolder) variants, which also showed improved expression rates and, in contrast to the wild type protein, they could be efficiently purified in their monomeric forms. Despite being considerably more stable, these engineered mutants largely preserved the wild type sequence (>98.8%). We demonstrate that the variant SF05 enabled the determination of the monovalent affinity between CD19 and a clinically approved FMC63-based CAR, as well as monitoring and phenotypic characterization of CD19-directed CAR-T cells in the blood of lymphoma patients. We anticipate that the SuperFolder mutants generated in this study will be highly valuable tools for a range of applications in basic immunology and CD19-targeted cancer immunotherapy.

## Introduction

In the past decade, immunotherapy has revolutionized our ability to treat cancer. One of the most extensively used antigens for cancer immunotherapy is the B cell-specific surface marker CD19, which was initially discovered as B4 antigen by using a B4 specific monoclonal antibody (mAb).^1,2^ Due to its highly defined expression pattern, being restricted to the B cell lineage, it serves as an optimal target for B cell malignancies, including acute lymphoblastic leukemia (ALL) and lymphomas. As a consequence, several highly potent CD19-targeting immunotherapies have been approved by the EMA and FDA, including the bispecific T cell engager (BiTE) blinatumomab and all four currently approved chimeric antigen receptor (CAR) T cell therapies.^3−8^

Due to its high relevance in immunology and immunotherapy, the availability of the soluble extracellular domain of CD19 (CD19-ECD) is of critical importance. In a pioneering study, De Oliveira et al. demonstrated the utility of the CD19-ECD for the detection of CAR-T cells.^9^ However, the CD19-ECD is a “difficult-to-express” protein that is prone to misfolding and aggregation and which only yields very low expression titers.^9−12^ These intrinsic properties of the CD19-ECD cause severe obstacles in the development and mechanistic analysis of CD19-directed immunotherapies. For example, it has been challenging, if not impossible, to study the interaction between monomeric CD19 and its binding partners. Oligomerization and aggregation of the CD19-ECD necessitated its expression on a cell surface, which precluded detailed analysis of a 1:1 interaction with another cell surface molecule, such as a CAR. Moreover, monomeric, high quality CD19-ECD protein would also facilitate directed evolution of CD19-targeting antibodies or alternative binding scaffolds, as well as affinity-maturations of existing antibodies or scFvs.^13−17^ Finally, a stable and functional CD19-ECD protein would also be a perfect tool for monitoring CAR-T cells in treated patients.^18^ Since clinically approved CAR molecules do not contain any tag for staining (to avoid potential immunogenicity), it has been challenging to detect them by flow cytometry. Specific labeling of CD19-directed CAR-T cells with their cognate antigen would allow multiparameter phenotyping by flow cytometry, quantification of CAR-T cell frequency, and analysis of CAR expression levels both in laboratory experiments and in patient samples.^18^ While an anti-idiotype antibody has been developed for the currently used CD19-specific CAR-T cells based on the FMC63 scFv, this antibody has not been publicly available.^18,19^ Moreover, an anti-idiotype antibody can only be used to detect one specific scFv, but it cannot be used as a general detection reagent for any CD19-specific CAR-T cell. Recently, commercial CD19 reagents have become available for the detection of CAR-T cells.^20,21^ Nevertheless, the lack of stable and, in particular, monomeric CD19-ECD protein remains a major limitation in the development, biochemical characterization, and monitoring of CD19-targeting immunotherapies.

We hypothesized that directed evolution of the CD19-ECD for improved folding and stability would be an efficient strategy to address these issues. Yeast surface display has been demonstrated to be a versatile tool for the selection of structurally improved protein variants in a high-throughput manner.^22^ Briefly, large, randomly mutated protein libraries are displayed on the surface of yeast cells, followed by selection for binding to structurally specific ligands, such as antibodies. This strategy has been applied successfully for the stabilization of several different types of proteins, including major histocompatibility complexes (MHCs),^23^ single-chain T cell receptors (scTCRs),^24^ and antigen-binding IgG1-Fc mutants (Fcabs).^25,26^

In this study, we isolated stable, natively folded CD19-ECD variants from a randomly mutated yeast display library. Those stabilized mutants showed improved folding, as measured with two different structurally specific antibodies and elevated yeast expression levels and thermal stabilities. Moreover, these properties translated to increased soluble expression titers in HEK293 cells and, in contrast to the wild type counterpart, also enabled the purification of a monomeric protein. We also show two exemplary applications of the final candidate termed SuperFolder 05 (SF05): (i) the analysis of the monovalent affinity of a CD19-directed CAR expressed on primary human T cells and (ii) the detection and phenotyping of CAR-T cells in patient samples. Taken together, we expect that the stabilized, monomeric CD19-ECD variants generated in this study will be highly useful tools to address various important questions and limitations in CD19-directed cancer immunotherapies, as well as in basic, mechanistic studies on this important B cell surface protein.

## Results

### CD19-ECD Lacking Exon 5 Shows Improved Folding

**Figure 1:**
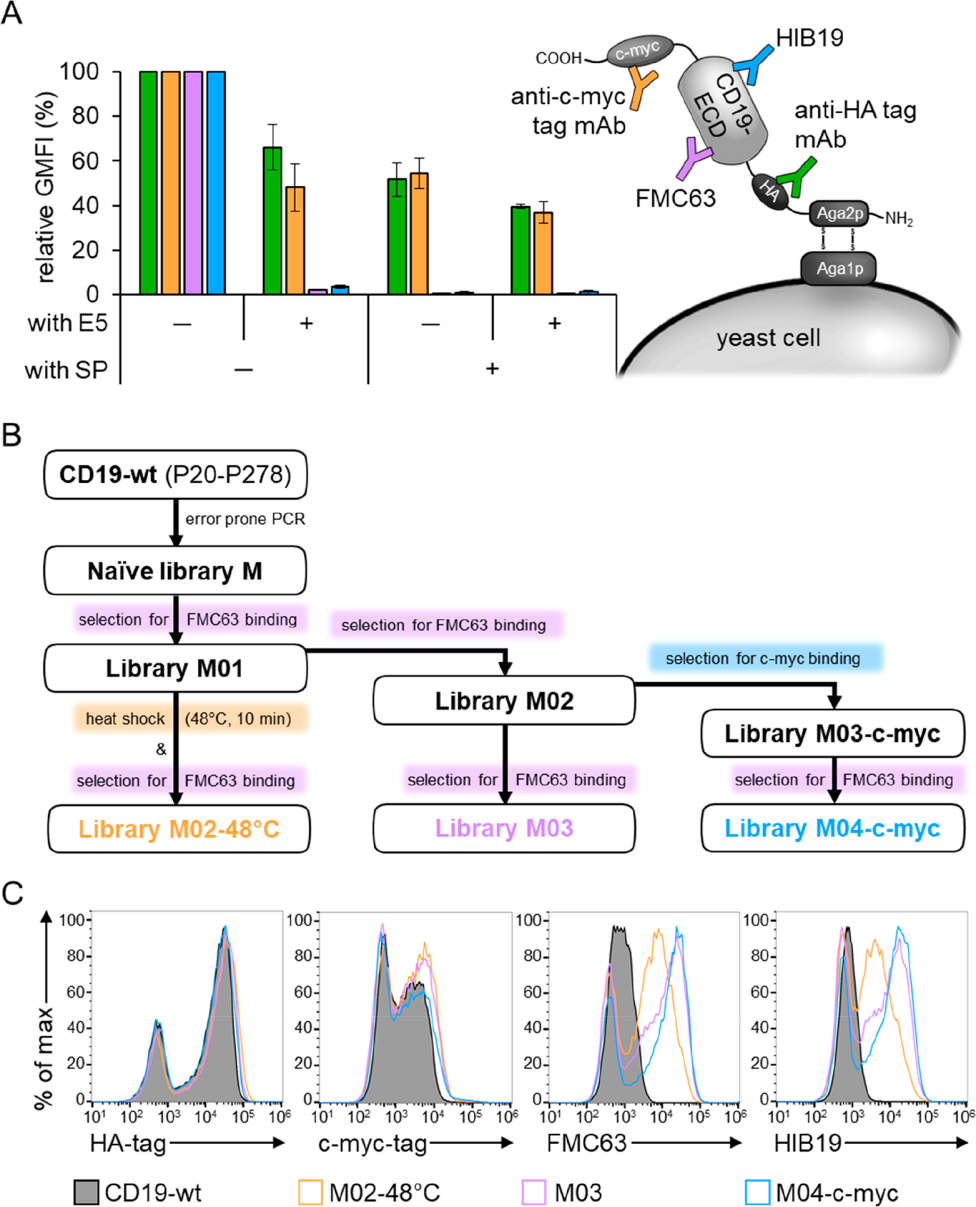
Preliminary yeast surface display experiment for library constructions and overview of the experimental strategy for selection of stabilized CD19-ECD variants. (A) Schematic overview of yeast displaying CD19-ECD with mAbs used for detection. Preliminary flow cytometric experiment probing four different yeast-displayed CD19-ECD constructs (with and without the signal peptide (SP) and exon 5 (E5), respectively) for binding to the antitag mAbs (anti-HA and anti-c-myc) and structure-specific anti-CD19-ECD mAbs (FMC63 and HIB19). Data were normalized to the version containing neither the SP nor E5. Averages ± SDs of the geometric mean fluorescence intensities (GMFIs) of three independent experiments are shown. (B) Schematic overview of the FACS selection strategy. The CD19-wt gene was used as template for error prone PCR, resulting in the library M. All following selection rounds included staining of cells with anti-HA mAb for normalization of expression level and either staining with FMC63 or the anti-c-myc mAb depending on the selection round and the strategy. In an alternative approach, library M01 was additionally subjected to a heat shock at 48 °C for 10 min prior to mAb staining. (C) Comparison of yeast displayed CD19-wt and the final library pools after selection. The surface displayed CD19-ECDs were probed for binding to the mAb panel, shown in (A) and analyzed by flow cytometry. One representative of three independent experiments is shown.

According to the Universal Protein Knowledgebase (UniProt KB), human CD19 (ID: P15391) is composed of in total 556 amino acids (aa), of which the first 19 residues are cleaved off as they represent the native signal peptide (SP). Based on this sequence analysis, the extracellular domain of CD19 is predicted to extend from P20 to K291. This part is encoded by exons 1−4 and 13 residues from exon 5 (E5).^27^ However, the fusion constructs reported in the literature encompass different lengths of CD19-ECD, for example, with and without the 13 residues from E5.^9−12,28^ Moreover, De Oliveira et al. designed two CD19-Fc fusion constructs comprising exons 1−4 or 1−3, respectively, of which only the construct containing the first four exons was able to specifically interact with FMC63-based CARs.^9^ To identify the most suitable starting construct for library construction, we tested CD19-ECD constructs containing either exons 1−4 only or additionally also the first 13 residues of E5. In addition, it has been reported that N-terminal signal peptides not only act as cellular address labels, but also modulate protein stability and aggregation during or after folding before they are cleaved off.^29−32^ To additionally analyze the effect of the native SP on CD19-ECD folding, both CD19-ECD lengths (with or without E5) were expressed on the surface of yeast in the presence or absence of the native SP. Expression levels were quantified via two tags: (i) an N-terminal hemagglutinin (HA) tag detecting total expression levels and (ii) a C-terminal c-myc tag indicating full length expression (Figure 1A).^13^ To directly analyze CD19-folding, binding to the two CD19-specific mAbs FMC63 and HIB19^33,34^ was analyzed. Of note, both mAbs showed strongly decreased binding after heat incubation of CD19-displaying yeast cells (Figure S1), demonstrating that they recognize a conformational epitope that is lost upon heat denaturation and thus they can be used as folding sensors. Analysis of the four different CD19-constructs clearly shows that the presence of E5 and the SP strongly reduced expression levels detected with anti-c-myc and anti-HA (Figure 1A). The effect on CD19 folding was even more pronounced, as demonstrated by nearly completely abolished binding of the structure-specific mAbs FMC63 and HIB19 (Figure 1A). Thus, the construct lacking both the E5 and the SP clearly showed the best folding properties and expression yield and was therefore chosen as the initial construct for library construction. This CD19-ECD is termed CD19 wild type (CD19-wt) in all further experiments.

### Selection of Stabilized CD19-ECD Variants

To engineer stabilized CD19-ECD variants, the CD19-ECD-wt gene was randomized by error prone PCR and displayed on the surface of yeast for high-throughput screening. Two yeast display libraries differing in mutation rates were generated (i.e., M and XL). Sequence analysis revealed 3.1 and 6.7 nucleotide mutations per CD19-ECD gene in the libraries M and XL, respectively (Figure S2A). Since a mutation frequency of approximately 2−5 base substitutions per gene has been recommended for directed evolution studies,^35,36^ the naïve library M was chosen for selection experiments. Importantly, all six types of nucleotide mutations were found (Figure S2B).

The theoretical diversity of all possible single nucleotide mutations in the CD19-ECD-wt gene is 2.3 × 10^3^ (777 bp, each of which can be mutated to three others), whereas the actual diversity of the naïve library M was determined to be 2 × 10^6^, thus, oversampling the theoretical diversity by almost 3 orders of magnitude. Therefore, even if the mutations are not perfectly evenly distributed, it can be assumed that almost every possible nucleotide mutation is present multiple times in the starting library.

To screen for improved folding and stability, the naïve library M was enriched for CD19 mutants that showed improved binding to the structure specific mAb FMC63, resulting in library M03. Alternative selection strategies included either a selection step for full length display using the C-terminal c-myc tag (library M04-c-myc) or a heat incubation step at 48 °C to increase the selection pressure toward thermal protein stability (library M02−48 °C), as described previously with other proteins.^36−38^ An overview of the different screening strategies is schematically summarized in Figure 1B.

The final libraries described above were characterized by flow cytometry and compared to CD19-wt displaying cells (Figure 1C). The peaks on the left represent nondisplaying yeast cells, which is typical for yeast surface display and provides an internal negative control.^13,39^ The displaying populations of CD19-wt show very little binding signal to the structure-specific antibodies FMC63 and HIB19, thus, merging with the nondisplaying peaks. In contrast, all three selected libraries show strongly improved binding to both antibodies with the displaying populations of libraries M03 and M04-c-myc shifting by more than an order of magnitude (Figure 1C). Remarkably, this trend is also observed for HIB19 recognition although this mAb was not used during the selection procedure. This strongly suggests that the fold of the CD19 variants was improved, otherwise the increased HIB19 signal could not be explained. Of note, expression levels were comparable to that of CD19-wt (HA- and c-myc-signals, Figure 1C), further supporting the hypothesis that the enhanced recognition by the structure-specific mAbs was not caused by increased expression levels, but by improved folding properties.

### SuperFolder (SF) CD19 Variants Reveal Native Fold and Higher Thermal Stability

**Figure 2:**
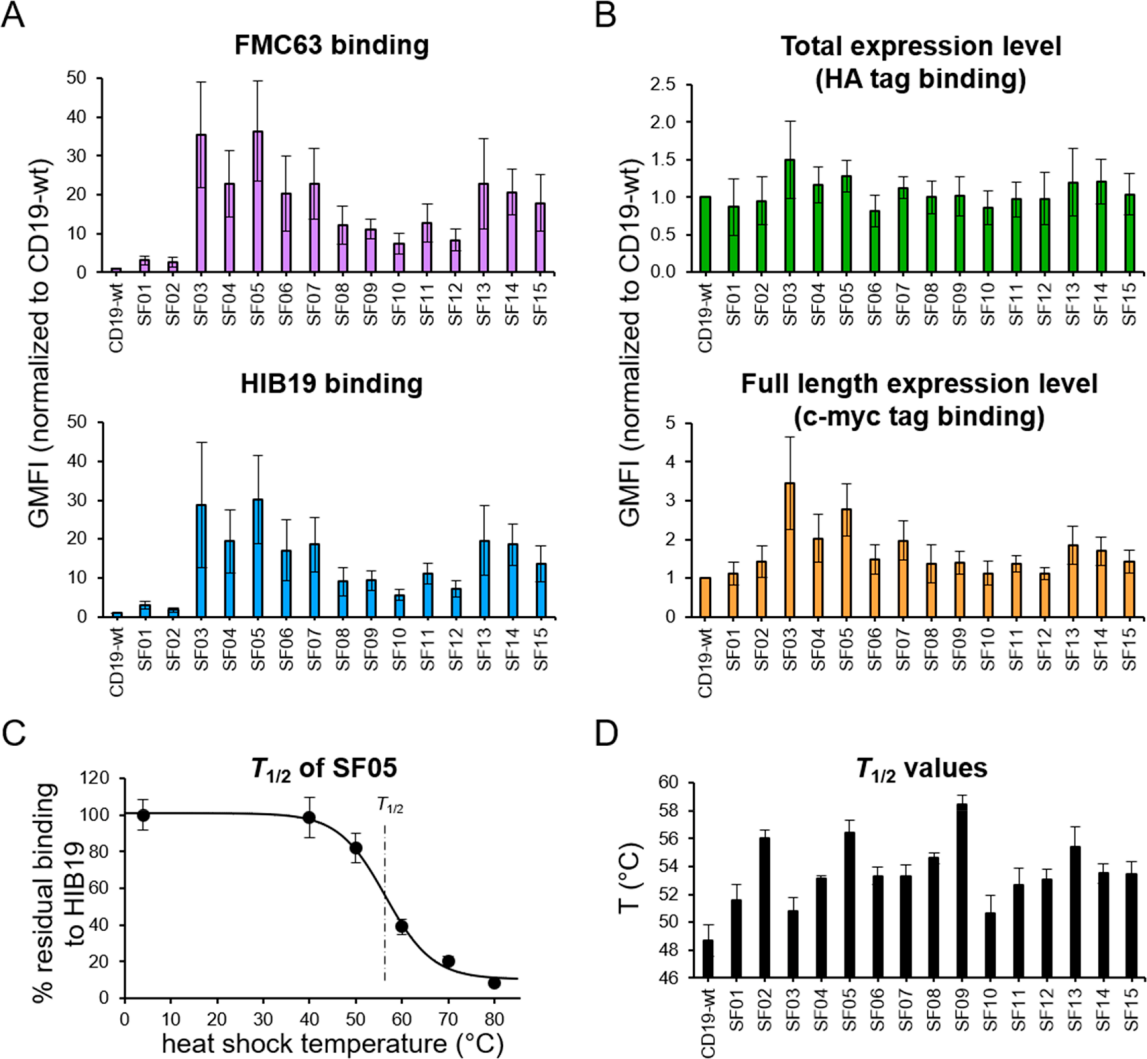
Enriched SuperFolder variants show improved structural integrity and thermal stability. Analysis of binding properties of selected SF variants (SF01-SF15) relative to CD19-wt. Displaying yeast cells were probed for binding to (A) the structure-specific anti-CD19-ECD mAbs (FMC63 and HIB19) and (B) the antitag mAbs (anti-HA and anti-c-myc) by flow cytometry. (C, D) Yeast displayed CD19-wt and SF variants were incubated at increasing temperatures for 10 min and subsequently analyzed for their binding to HIB19 by flow cytometry. The residual binding of HIB19 is plotted vs the incubation temperature. Resulting data allow the calculation of *T*_1/2_ values based on modeled denaturation curves.^38,40^ (C) This diagram depicts a representative curve for SF05 normalized to the mean of nonheated sample. (D) The plot shows calculated *T*_1/2_ values of CD19-wt and SF variants. All data in this figure represent averages ± SDs of three independent experiments.

A total of 32 clones of each library (i.e., M02−48, M03, and M04-c-myc) were sequenced, yielding a total of 23 individual sequences, of which 15 clones (termed SuperFolder 01−15) were chosen for further analysis. All SuperFolder (SF) mutants were displayed on yeast individually and probed for binding to the structure-specific mAbs FMC63 and HIB19 (Figure 2A). Besides SF01 and SF02, which are only slightly improved compared to CD19-wt, all remaining SF variants (i.e., SF03-SF15) showed a 7−36-fold rise in binding signal to the structure-specific mAb FMC63 when compared to CD19-wt. Importantly, highly similar trends were observed with the second mAb HIB19 (Figure 2A), again demonstrating that the increase of the binding signal is mediated by improved folding properties, since HIB19 was not used for selection. In other words, the SF mutants, which were screened for elevated FMC63-binding, also showed enhanced interaction with HIB19.

In addition, expression on the yeast surface was analyzed with antibodies recognizing the two expression tags HA and c-myc. Total expression as measured with anti-HA was comparable between all mutants (Figure 2B), whereas the full-length expression analyzed with anti-c-myc was elevated for some SF variants. The most highly expressed clones were SF03 and SF05 with ∼3-fold increases compared to CD19-wt (Figure 2B). Nevertheless, this slightly elevated expression level only partially explains the dramatic improvement of the binding signal toward both structure specific mAbs (Figure 2A), again suggesting that the main driver is an improved CD19-ECD fold. It should also be noted that the sequences of all 15 SF variants are still very similar to that of CD19-wt with only 1−5 amino acid substitutions, as will be further discussed below.

Since the integrity of a protein fold is ultimately linked to its thermal stability, the temperature of half-maximum irreversible denaturation (*T*_1/2_) was also determined on the surface of yeast, as exemplarily shown for SF05 in Figure 2C. It has been shown that the *T*_1/2_ values of yeast-displayed proteins strongly correlate with the thermal stabilities of their soluble counterparts.^38,40,41^ Remarkably, all analyzed SF mutants were stabilized compared with CD19-wt. SF09 and SF05 were the two most stable mutants with *T*_1/2_ values of 58.4 and 56.4 °C, respectively, as compared with 48.7 °C for CD19-wt (Figure 2D). Together, these data demonstrate that the enriched SF variants show a considerably increased stability, as well as improved folding properties as measured with two different structure specific antibodies.

### Location of Enriched Mutations in the CD19-ECD Structure

**Figure 3:**
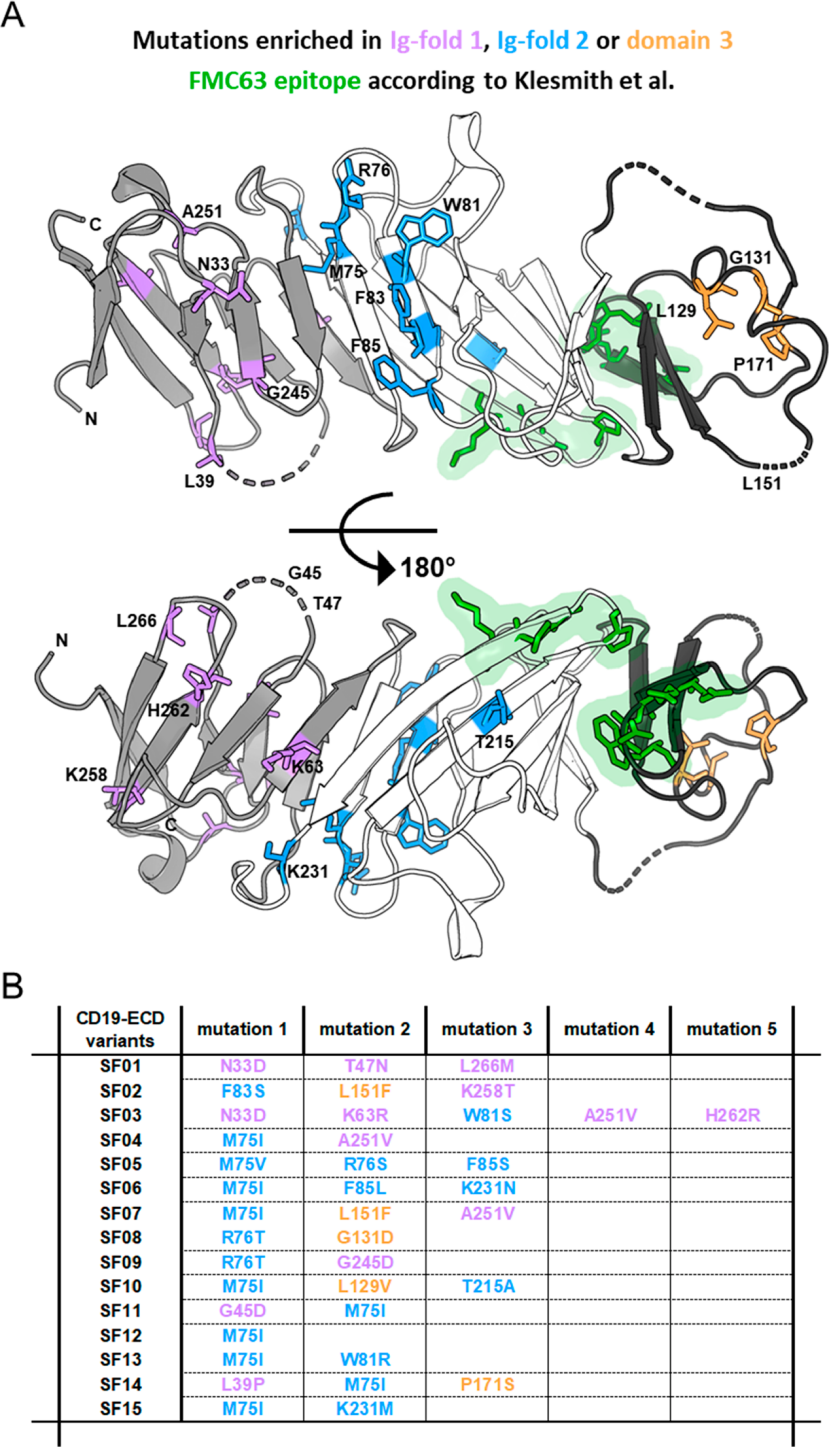
Location of SuperFolder mutations in the CD19-ECD structure. (A) Cartoon representation of CD19-ECD (PDB ID: 6AL5) created by using PyMOL Molecular Graphics System, Version 2.4.0a0, Schrödinger, LCC. The structure is colored according to its topology.^12^ The two Ig-like domains (domain 1: E21-P69, P235-R277 and domain 2: G70-G117, Q186-R234) are colored in gray and white, respectively. The connecting third semistructured domain 3 (S118−S185) is colored in black. The loops that were not resolved within the crystal structure are shown as dashed lines. Residues of mutated positions are shown as sticks and colored according to the topology in purple (Ig fold 1), blue (Ig fold 2), and orange (domain 3). Green highlighted residues represent the FMC63 epitope (W159, D162, R163, E165, P219, G221, P222, K223) according to Klesmith et al.^42^ (B) Mutations of selected variants (SF01-SF15) that were individually analyzed on the surface of yeast. Mutations are colored according to the structure shown in (A).

Contrary to previous assumptions, where the CD19-ECD was predicted to be composed of a tandem of two Ig-like domains separated by a non-Ig-like domain,^1,27^ the recently solved X-ray structure revealed a new unique topology.^12^ The ECD is composed of a swapped arrangement of both Ig-like domains (domain 1: E21-P69, P235-R277 and domain 2: G70-G117, Q186-R234) in addition to a connecting semistructured domain (domain 3: S118−S185; Figure 3A). That is, in the primary structure, the first half of domain 1 is separated from its second half by domains 2 and 3. Similarly, the first half of domain 2 is separated from its second half by domain 3. In general, all mutations of SF01−15 are distributed over the entire extracellular domain of CD19 with a mutational hot spot at residues 75 and 76 (Figure 3A). This hot spot region is spatially separated from the known FMC63 epitope (Figure 3A).^42^ In general, none of the mutations enriched in our SF mutants overlapped with the FMC63 epitope, thus, providing further evidence that the substitutions selected in our yeast display experiments improve protein structure and stability, but not FMC63 binding. Of note, all SF mutants except for SF03 contain only 1−3 amino acid substitutions (Figure 3B). With a total length of 259 amino acids, this corresponds to 98.8−99.6% wild type sequence, demonstrating that, despite their significant structural improvement, they are still highly similar to the original protein.

### SuperFolder CD19 Variants Yield Increased Soluble Expression Titers and Monomeric Protein

**Figure 4:**
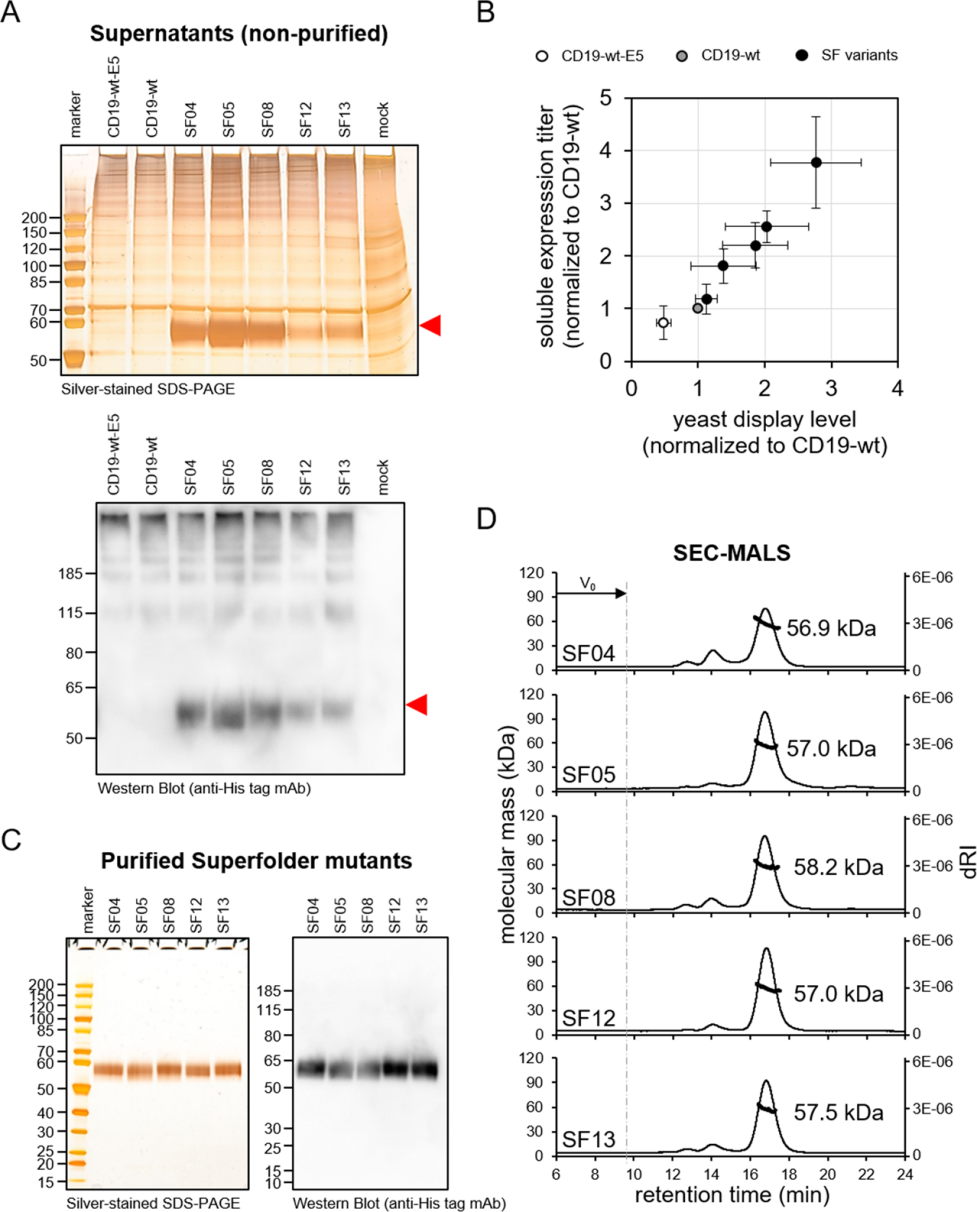
Soluble expression of SuperFolder variants yields monomeric protein. (A) Analysis of the supernatant of HEK293-6E cells transiently transfected with plasmids encoding CD19-wt, CD19-wt-E5, or selected SF variants (i.e., SF04, 05, 08, 12, 13) by silver-stained SDS-PAGE and Western Blot detecting His-tagged protein. Mock (negative control) refers to cells that were transfected with sterile H_2_O instead of plasmid DNA. The red marks indicate migration of the monomeric CD19-ECD protein. (B) Correlation between the expression level of yeast surface displayed proteins (detection via c-myc tag) with corresponding solubly expressed formats from HEK293-6E supernatants (detection via His-tag using BLI). Shown are CD19-wt-E5 (white), CD19-wt (gray), and all solubly expressed SF variants (i.e., SF04, 05, 08, 12, 13; shown in black). Data were normalized to CD19-wt. Averages ± SDs of three independent experiments are shown. (C) Analysis of 500 ng of each SEC-purified SF variant by silver-stained SDS-PAGE and Western Blot detecting His-tagged protein. (D) SEC-MALS analysis of purified SF variants. A Superdex 200 10/300 GL column was loaded with 25 μg of protein upstream to MALS analysis. The molecular mass of the monomeric protein peak of the respective SF variants was calculated based on the light scattering data. *V*_0_ indicates the elution time of the void volume. One representative of two independent experiments is shown.

Next, we investigated whether the improved structural integrity observed on the surface of yeast translates to enhanced folding properties of their soluble counterparts. Five SF mutants (SF04, SF05, SF08, SF12, and SF13) were chosen based on their binding properties to the two structure-specific mAbs, their thermal stability, as well as the location and number of mutations, which was limited to three per mutant. We also included SF12, which only contains a single amino acid mutation. Moreover, the wild type version of CD19-ECD was also expressed solubly, both with (CD19-wt-E5) and without (CD19-wt) exon 5, since both versions have been described in the literature.^9−12,28^ All proteins were expressed as His-tagged SUMO-CD19-fusions in HEK293-6E cells, followed by analysis of the supernatants. Remarkably, whereas both wild type versions (with and without E5, respectively) only yielded oligomeric and aggregated protein, monomeric bands were observed for all five SF variants, as assessed by SDS-PAGE and Western Blot analysis (Figure 4A). In particular, SF04, SF05, and SF08 showed strong bands at the expected size of monomeric protein (44 kDa polypeptide with five putative N-glycosylation sites, contributing ∼2−3 kDa each,^43^ yielding a total expected molecular mass of ∼55−60 kDa; Figure 4A). Interestingly, the expression titers obtained after soluble expression in HEK293-6E cells strongly correlated with the full-length expression levels measured on the yeast surface (Figure 4B). Of note, all five SF mutants were expressed at higher levels than the two wild type versions, with the highest expression levels for SF05 (Figure 4B).

Next, the SF variants were purified by affinity chromatography via their His-tag, as well as size exclusion chromatography (SEC), yielding monomeric bands in subsequent SDS-PAGE and Western Blot analyses (Figure 4C). Since both wild type versions were almost exclusively oligomerized or aggregated, purification of monomeric protein was not possible in these cases. Final analysis of the purified SF variants by SEC confirmed that the proteins were mostly present as monomers (∼57 kDa), as determined by multiangle light scattering (MALS) analysis. Only small fractions of presumably dimeric and trimeric proteins were observed, and large molecular aggregates were not detectable at all (Figure 4D).

To sum up, these data clearly show that the SF variants, which have been screened for improved structural integrity by yeast surface display, also show elevated expression titers when expressed solubly in HEK293-6E cells and, importantly, they yield monomeric protein, which could not be obtained with their nonmutated wild type counterpart. Overall, SF05 proved to be the best variant. It was strongly stabilized (8 °C increase in *T*_1/2_, Figure 2D), showed highly elevated binding to both structure specific antibodies (Figure 2A) and yielded almost exclusively monomeric protein after HEK293-6E expression and subsequent purification (Figure 4D). Therefore, SF05 was chosen for all further experiments.

### Analysis of the Monovalent CAR-CD19 Affinity

**Figure 5:**
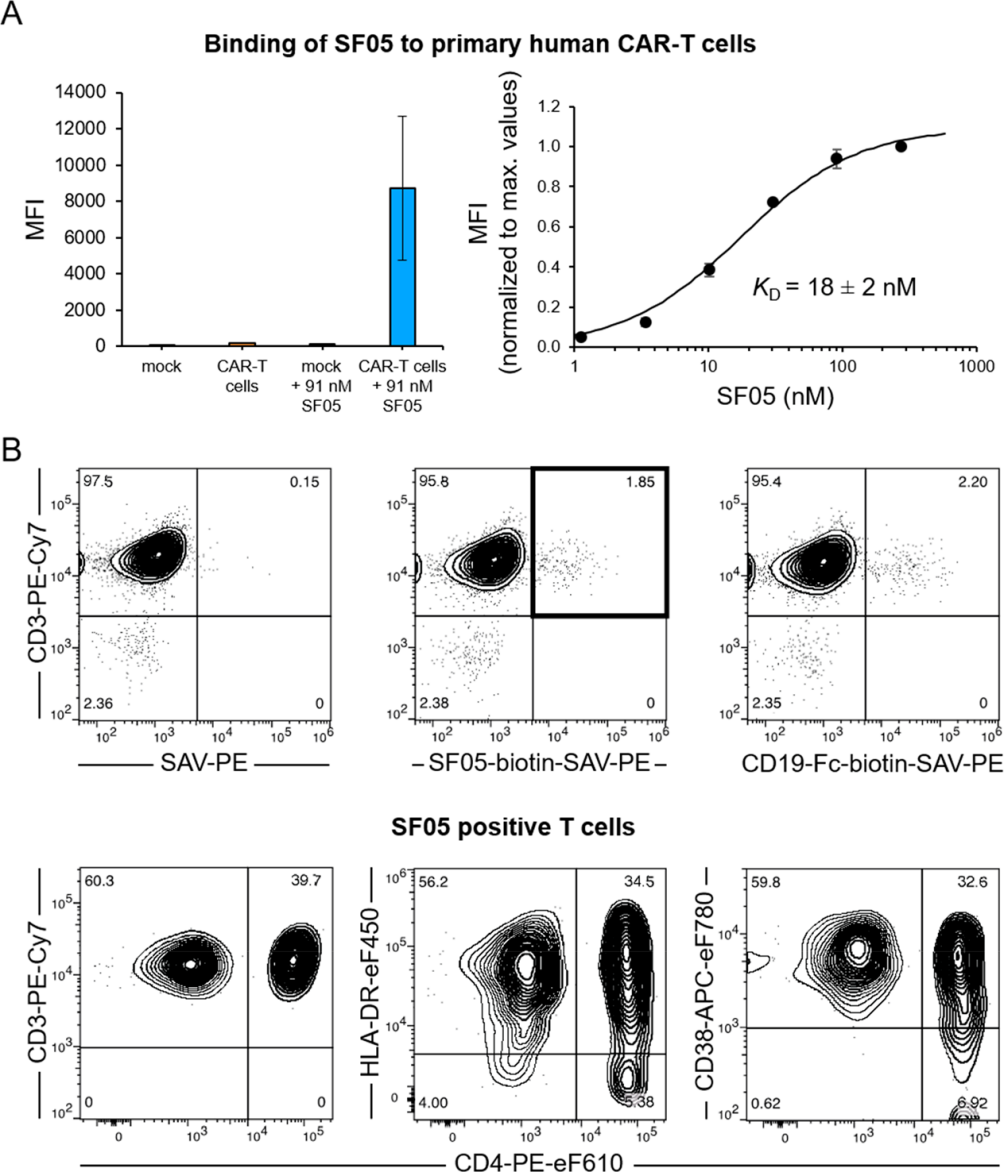
Analysis of the monovalent affinity of FMC63-CARs and monitoring of CAR-T cells in patients. (A) CD19-directed CAR-T cells based on the scFv FMC63 were incubated with increasing concentrations of SF05-AF647 and analyzed by flow cytometry. The diagram on the left shows a comparison of SF05-stained CAR-T cells with various controls as indicated. By fitting a one-set-of-sites binding model to the data from the titration of SF05-AF647 on CAR-T cells, a binding isotherm was obtained,^44^ yielding a *K*_D_ value of 18 ± 2 nM (diagram on the right). Two independent experiments with T cells from three different donors; averages ± SDs. (B) Shown are CAR patient’s CD3 positive T cells coexpressing the CD19 CAR as detected by SF05 (upper row, middle plot) or the commercially available CD19-Fc detection reagent (upper row, right plot), both conjugated to biotin, in comparison to sham-stained T cells (upper row, left panel). Lower panel shows subset distribution of SF05-identified T cells upon CD4 staining (left panel) and their activation status upon HLA-DR and CD38 counter staining (middle and right plot), respectively. Markers were set according to negative control populations, numbers in quadrants indicate percent positive cells. One representative of three independent experiments is shown.

Next, we investigated whether SF05 allows for detection of CD19-directed CAR-T cells. For that purpose, primary human T cells were transduced with the clinically approved FMC63-based CAR construct of tisagenlecleucel (Kymriah) and subsequently incubated with SF05, yielding a robust shift of the entire CAR-T cell population. Importantly, mock-transduced T cells incubated with SF05 were almost indistinguishable from unstained cells, demonstrating that SF05-binding was highly specific and CAR-dependent (Figure 5A).

Despite the high clinical relevance of the FMC63 scFv, it has been a challenge to determine its affinity to CD19 when expressed in a CAR format. However, the monomeric nature of the SF05 variant enables the analysis of its monovalent affinity to FMC63-based CARs. Titration of SF05 on primary human CAR-T cells yielded an affinity of 18 ± 2 nM (Figure 5A), which is in line with other studies that reported values between 6 and 47 nM for the monovalent interaction between wild type CD19 and the FMC63 scFv.^11,45,46^

### Monitoring of CAR-T Cell Patients with SF05

Based on these promising results, we also tested whether SF05 would reliably detect CAR-T cells *in vivo*, that is, in the bloodstream. For this purpose, the peripheral blood of a number of CAR-T cell recipients suffering from diffuse large B cell lymphoma and receiving tisagenlecleucel (Novartis, Switzerland) was stained with SF05 and counterstained with a collection of monoclonal antibodies against various leukocyte differentiation and activation antigens. When gating on lymphocytes and excluding monocytes and granulocytes via dump channels for the lineage markers CD14 and CD15, respectively, the population of CD19 CAR-T cells could be clearly discriminated within the CD3-positive T cells by double staining for CD3 and SF05 (Figure 5B). In comparison to a recently introduced commercially available CD19-Fc CAR detection reagent, similar frequencies of CAR-T cells could be detected, which confirmed the results of the SF05 staining. Similar data were obtained by analyzing blood collected from the same CAR-T cell patient on consecutive days (data not shown). The counterstaining of the T helper cell marker CD4 showed that both CD4-positive and CD4-negative (i.e., presumably CD8-positive cytotoxic) T cells were among the CAR-T cells identified with SF05. Furthermore, the activation status of the SF05-identified CAR-T cells could be clearly determined by analysis of HLA-DR and CD38 expression. These analyses suggest that the non-CD4 (i.e., mostly CD8-positive) T cells were more strongly activated than the CD4 T cells in the respective patient. In summary, SF05 is ideally suited to detect and track CD19 CAR-T cells *in vivo* and, in combination with other markers, to determine their differentiation and activation status.

## Discussion

In this study we generated CD19-ECD mutants by screening a large combinatorial CD19-ECD library (two million variants) for binding to the conformation-specific antibody FMC63. Of note, although the yeast displayed-CD19 library was screened for improved interaction with FMC63, the obtained variants also showed increased binding to the structurally specific antibody HIB19, demonstrating that the selection pressure was directed toward protein folding and stability instead of enhanced affinity to FMC63. This hypothesis is further supported by the observation that the mutational hotspot identified in this study and all mutations of the final mutant SF05 are distant from the FMC63-epitope (Figure 3A), which has been shown to be an immunodominant epitope recognized by most CD19-reactive mAbs (also including HIB19).^42,47^

Importantly, the obtained CD19 mutants combine several beneficial properties: (i) elevated thermal stability, (ii) improved folding as shown by enhanced binding to both structurally specific antibodies, (iii) enhanced expression levels, and (iv) increased prevalence of the monomeric fraction. Consistent with other reports in literature,^24,37^ increased yeast surface display levels strongly correlated with elevated soluble expression titers from mammalian cells (Figure 4B). In addition to the increased total expression level, a significant fraction of the protein was monomeric, which was in stark contrast to CD19-wt, which almost exclusively yielded aggregated or oligomerized protein. In fact, the lack of significant amounts of nonaggregated CD19-wt even precluded the purification of monomeric CD19-wt. This combined effect of increased total expression with an elevated fraction of monomeric protein is also reflected by the decent yield of purified functional SF05 protein in this study (∼8 mg L^−1^) without any optimization, despite the fact that the CD19-ECD is considered a highly “difficult-to-express” protein.^11,18^ Most importantly, the final purified SF05 product is almost exclusively monomeric (Figure 4D), which, to the best of our knowledge, has not been described with any other CD19-ECD version before.

In previous reports, the CD19-ECD was expressed either in the presence or absence of the first 13 amino acids of exon 5. Our yeast display data clearly demonstrate that this largely hydrophobic part is highly detrimental to native folding and therefore we excluded this stretch from all further CD19 constructs.

The mutations enriched during the directed evolution experiment were broadly distributed over the entire CD19-ECD (Figure 3A). None of the enriched mutations affected or generated an N-glycosylation site or a cysteine position. Of note, a mutational hot spot was identified at positions M75 and R76, where all clones except for SF01, SF02 and SF03 contain a mutation (Figure 3B). Interestingly, two of these three SF mutants without any mutation at this hot spot (i.e., SF01 and SF02) also showed the lowest binding signal to both structurally specific antibodies, supporting the hypothesis that those hot spot mutations are highly beneficial for proper folding of the CD19-ECD. Mutants containing only one mutation at either pos. 75 or 76 already showed considerably improved folding and thermal stability (Figure S3A,B). The top performing single mutant R76T showed an increase in thermal stability of 8 °C, which also translated to a significant fraction of monomeric protein after soluble expression in HEK293 cells (Figure S3C). Together, these data suggest that the CD19 sequence at these two positions is suboptimal, and mutations lead to significant structural improvements.

Interestingly, during the revision process of this manuscript, the CD19−CD81 complex was resolved by cryo-EM,^48^ demonstrating that the mutational hot spot identified in the present study (and, in fact, all three mutations of SF05) is located in the CD81 epitope. It is known that CD81 is necessary for efficient delivery of CD19 to the cell surface.^48,49^ Thus, our data combined with the recent CD19−CD81 structure suggest that, in the absence of CD81, the respective unoccupied surface of CD19 is prone to partial misfolding and aggregation, thus, demanding mutational optimization in our yeast display-based directed evolution experiments.

During our study, another report was published by Klesmith et al., where the CD19-ECD was also engineered using yeast surface display.^28^ These authors fused the engineered CD19-ECD to an antigen-binding domain, which enabled redirection of CD19-specific CAR-T cells to other antigens. Apart from this fundamentally different application, Klesmith and colleagues also employed another engineering strategy, which combined various libraries and selection approaches directed toward improved stability, protease resistance and FMC63 binding, ultimately yielding a CD19-ECD version (termed CD19.1) containing 26 mutations. This extensively mutated variant showed strongly elevated binding signal to FMC63, but its thermal stability was increased by only 3 °C. In contrast, the SF variants generated in our study only contained limited numbers of mutations. Besides SF03, which was not chosen for soluble expression and detailed characterization, all other SF mutants contained a maximum of three mutations (Figure 3B). Nevertheless, these SF mutants were stabilized by up to 10 °C. Thus, the mutants engineered in the present study largely preserve the wild type sequence, but are considerably stabilized.

Given the use of the FMC63 scFv in clinically approved CAR-T cells, a detailed understanding of the molecular mechanism including the monovalent affinity of the FMC63 scFv incorporated into a CAR is crucial. However, the aggregation-propensity of the CD19-ECD would result in avidity effects when analyzing its affinity toward CAR-T cells, which is expected to be further enhanced by the dimeric nature of currently used CAR molecules.^50^ Thus, the determination of the true monomeric affinity of this clinically highly relevant interaction has been challenging. In this study, the monomeric behavior of SF05 combined with its limited number of mutations enabled the analysis of a reliable monovalent affinity to FMC63-based CARs expressed on the surface of primary human T cells, yielding a *K*_D_ of 18 ± 2 nM. This value is in good agreement with other interaction measurements using CD19-wt and FMC63 scFv (6 to 47 nM).^11,45,46^ Importantly, to the best of our knowledge, this is the first affinity measurement between strictly monomeric CD19-ECD and FMC63-based CARs expressed in primary human T cells.

Another important application of a stable and natively folded CD19-ECD is the detection of CAR-T cells in patient samples. In the case of CD19-directed CAR-T cells, this task has been an important limitation due to the paucity of stable, soluble antigen for flow cytometry analysis. Therefore, in many studies, quantitative real-time PCR (qPCR) has been employed to estimate the prevalence of CAR-T cells. It should be noted that due to its high sensitivity,^51,52^ qPCR is the method of choice for many lymphoma studies, where the majority of the CAR-T cells are not found circulating in peripheral blood, but rather in secondary lymphoid compartments such as lymph nodes and the spleen. Nevertheless, even large clinical studies such as JULIET, treating diffuse large B cell lymphoma patients with CAR-T cells succeeded in reliably monitoring CAR-T cells circulating in peripheral blood by flow cytometry.^53^ Importantly, qPCR does not enable phenotypic characterization of the CAR-T cells. Moreover, qPCR only measures the abundance of the inserted CAR vector, but not CAR expression levels, which are known to be influenced by many parameters including the promoter used, the insertion site in the genome, the number of copies per genome, chromatin accessibility, antigen-mediated CAR down-regulation, among others.^18^ For example, it has been shown that the semirandom integration of lentivirus- or retrovirus-based vectors yields a broad distribution of expression levels,^54^ which cannot be analyzed by qPCR. In contrast, flow cytometric analysis of patient samples enables phenotypic characterization of CAR-T cells by using multicolor panels, as well as analysis of CAR expression density. Therefore, anti-idiotype antibodies have been developed, which allow for specific staining of CAR-T cells harboring the scFv FMC63.^19^ However, apart from limited availability of those anti-idiotype antibodies,^18^ they can only be applied for a specific type of scFv. Therefore, the best solution to the problem of CAR-T cell detection would be a stable and easy-to-use antigen that specifically interacts with the CAR molecule.^18^ Recently, a CD19-Fc chimeric protein has been commercially released, allowing detection of CAR-T cells.^20,21^ Nevertheless, the SF05 variant generated in this study represents an Fc-free and monomeric alternative, as demonstrated by the phenotyping of CAR-T cells in patient samples (Figure 5B).

Together, in this study we generated stabilized versions of the immunologically and clinically highly important CD19-ECD protein. Although the final mutant SF05 contained only three point mutations among 259 amino acids, it was considerably more stable than its wild type counterpart, which also translated to improved expression yields in yeast and mammalian cells and to a strongly increased fraction of monomeric protein, which could be efficiently purified. We expect that the CD19-ECD mutants engineered in this study will be highly valuable tools for various scientific areas, including basic B cell immunology and cancer immunotherapy.

## Materials and Methods

### Selection of a CD19-ECD Construct for Library Construction

To identify the optimal CD19-ECD as initial construct for library construction, four different constructs encompassing exon 1−4 (SP- E5-, P20−P278) and either the native signal peptide “MPPPRLLFFLLFLTPMEVR” (SP+ E5-, M1-P278) or the 13 residues from exon 5 (SP- E5+, P20-K291) or both (SP+ E5+, M1-K291) were displayed on the surface of yeast. Therefore, the respective insert was amplified from the pL vector containing the ECD of CD19 including the native SP and exon 1−5^11^ and subcloned into the pCT-CON2 vector, resulting in the following fusion construct: Aga2p−HA tag−(G_4_S)_3_ linker−CD19-ECD−c-myc tag (Figure 1A).^13,39^ Transformation of *Saccharomyces cerevisiae* (strain EBY100) with plasmid preparation of the pCT-CON2 encoding the respective CD19-ECD was performed using the Frozen-EZ Yeast Transformation II Kit (Zymo Research, U.S.A.) according to the manufacturer’s instructions. Transformed yeast were plated on SD-CAA agar plates and incubated at 30 °C for 4 days.

#### Yeast Display Experiments

All yeast display experiments were performed in triplicate, as described previously.^38,55^ Briefly, cultures were grown to the stationary phase in SD-CAA medium overnight at 30 °C. The next morning, cultures were diluted to an OD_600_ of 1 in SD-CAA medium. After 5−6 h, cells were centrifuged, and yeast surface expression was induced by resuspending the cells in SG-CAA medium and shaking overnight at 20 °C. All subsequent procedures were performed in PBSA (PBS + 0.1% bovine serum albumin) and the centrifugation steps were conducted at 2000 g for 3 min. All staining steps were performed at 4 °C for 30 min, shaking. The next day, the cells were washed and resuspended in PBSA to achieve an OD_600_ of 1. For heat shock experiments, the cell suspensions were aliquoted in 500 μL portions in microcentrifuge tubes, chilled on ice for 10 min followed by incubation at the respective temperature (i.e., ice, 45, 55, 65, or 75 °C) for 10 min in a thermomixer (Eppendorf, Germany). After cooling on ice, several 25 μL of each aliquot were transferred to a conical-bottom 96-well-plate (Thermo Fisher Scientific, U.S.A.). For FACS experiments cells were stained with anti-HA AF647 mAb (clone 16B12, BioLegend, U.S.A.) at a concentration of 10 nM. All following staining reagents were used at a concentration of 67 nM. For detection of the full-length expression level, cells were incubated with mouse anti-c-myc mAb (clone 9E10), followed by incubation with goat antimouse AF488 IgG (both from Thermo Fisher Scientific, U.S.A.). For analysis of the proper folding of the respective CD19-ECD, cells were stained with two different conformation-specific anti-CD19 mAbs. Unlabeled rabbit anti-CD19 mAb (clone FMC63, Absolute Antibody Ltd., U.K.) was detected with antirabbit F(ab’)2 Fragment AF488 (Cell Signaling Technology, U.S.A.) and PE-labeled anti-CD19 mAb (clone HIB19, BioLegend, U.S.A.) was used in a single staining step. Following a terminal washing step, cells were analyzed either on a FACSCanto II (BD Biosciences, U.S.A.) or a Gallios Flow Cytometer or a CytoFLEX S (both from Beckman Coulter, U.S.A.). During flow cytometric measurements, 3000 events (singlets gate) were recorded and each cell pool was gated for the desired morphology and presence of single cells only. Resulting data were analyzed using the FlowJo software (BD Biosciences, U.S.A.).

#### Library Construction and Characterization

Based on the previous results, the pCT-CON2 vector containing CD19-ECD encompassing exon 1−4 (P20−P278) was used as template for error prone PCR using the GeneMorph II Random Mutagenesis Kit (Agilent Technologies, U.S.A.). By using different amount of template DNA, four libraries were constructed (i.e., S, M, L, and XL). The respective mutation frequencies were estimated based on the quantification of the PCR product yield. Gel-purified inserts from two libraries (i.e., M and XL) were PCR amplified using a *Taq* DNA Polymerase (New England Biolabs, U.S.A.) and purified via ethanol precipitation. EBY100 was transformed with the respective inserts together with the linearized pCT-CON2 vector backbone, both containing homologous regions, using the lithium acetate method.^55^ After growing both EBY100 libraries in SD-CAA medium at 30 °C for 48 h, pCT-CON2 plasmid DNA was isolated using the Zymoprep Yeast Plasmid Miniprep II Kit (Zymo Research, U.S.A.) according to the manufacturer’s instructions and transformed into 10-beta Electrocompetent *Escherichia coli* (New England Biolabs, U.S.A.) for sequencing of 48 clones per library.

### FACS Procedure for Selection of Stable CD19-ECD Variants

Based on the mutation frequencies per CD19-ECD gene, the naïve library M was chosen for selection of stable CD19 variants. Thus, the library M was cultured overnight in SD-CAA medium, followed by induction of yeast surface display as described above or elsewhere.^38,55^ To normalize on yeast surface expression level, all rounds of sorting included staining of yeast cells with the anti-HA AF647 mAb (clone 16B12, BioLegend, U.S.A.). Within the first round of sorting, yeast cells were stained with unlabeled rabbit anti-CD19 mAb (clone FMC63, Absolute Antibody Ltd., U.K.) followed by detection using an antirabbit F(ab’)2 Fragment AF488 (Cell Signaling Technology, U.S.A.). Positive cells were selected by FACS resulting in the library pool M01. This pool was split, and one part was sorted in the same way as before, yielding the library pool M02. Since it was shown that thermal stability of the surface expressed proteins strongly correlates with the stability of the corresponding soluble form, the other part of M01 was subjected to a heat shock at 48 °C for 10 min prior FMC63 staining resulting in the library pool M02−48 °C.^36−38^ Library M02 was split and screened in parallel for increased FMC63 and c-myc (staining of cells with anti-c-myc AF488 mAB (clone 9E10, Thermo Fisher Scientific, USA)) binding to gain the library pools M03 and M03-c-myc, respectively. The additional sorting round for full length expression was included due to the presence of the small number of frameshifts within the naïve library. As final sorting round, the library M03-c-myc was again selected for improved folding using FMC63 staining, resulting in the library pool M04-c-myc. Figure 1B shows a detailed overview of the selection procedure for directed evolution of CD19-ECD. Cells were either sorted on a MoFlo Astrios EQ Cell Sorter (Beckman Coulter, U.S.A.) or on a FACSAria Fusion (BD Biosciences, U.S.A.). After in total 2−4 rounds of sorting, every library was individually tested for binding to the antibody panel described above. Based on those results, the plasmid DNA of libraries M02−48 °C, M03, and M04-c-myc was isolated and used for *E. coli* transformation. A total of 32 clones per library were sequenced. Fifteen CD19-ECD mutants (SuperFolder 01−15) were recloned into *S. cerevisiae* using the Frozen-EZ Yeast Transformation II Kit (Zymo Research, U.S.A.) and analyzed for binding to the above-described antibody panel. Additionally, the binding to PE-labeled anti-CD19 mAb (clone HIB19, BioLegend, U.S.A.) after subjecting the yeast displaying cells to a heat shock at 40, 50, 60, 70, and 80 °C for 10 min was analyzed. Obtained data yield a denaturation curve that allows the calculation of the temperature of half-maximal irreversible denaturation (*T*_1/2_) for CD19-wt and each SuperFolder (SF) mutant, as previously described.^38,40^

The single point mutations for the plasmid preparation of the single mutants were introduced using the QuikChange Lightning Site-Directed-Mutagenesis kit (Agilent Technologies, U.S.A.) according to the manufacturer’s instructions and the pCT-CON2 vector containing the CD19-wt fusion construct as template. *S. cerevisiae* was transformed, yeast surface display was induced, single mutants were analyzed for mAb binding and *T*_1/2_ values were determined as described above.

### Expression and Purification of SuperFolder Mutants

The five most promising SF mutants (i.e., SF04, 05, 08, 12, 13) and CD19-wt were chosen for soluble expression in HEK293-6E cells (NRC Biotechnology Research Institute, Canada).^56^ Cells were cultivated in FreeStyle F17 expression medium (Thermo Fisher Scientific, U.S.A.) supplemented with 0.1% Kolliphor P188 (Merck, Germany), 4 mM l-glutamine (Carl Roth, Germany) and 25 μg mL^−1^ G418 (Biochrom, Germany) in 50 mL TubeSpin bioreactor tubes (TPP Techno Plastic Products AG, Switzerland) at 37 °C, 80% humidity, 7% CO_2_, and 220 rpm in a Climo-Shaker ISF1-XC (Adolf Kühner AG, Switzerland). High quality plasmid preparations of the pTT5 vector (NRC Biotechnology Research Institute, Canada) containing the coding sequence for the IgGκ signal peptide,^57^ followed by an 8× histidine tag, an AviTag,^58^ the small ubiquitin-like modifier- (SUMO) star fusion protein,^59^ a human rhinovirus (HRV) 3C cleavage site and either CD19-wt or the SF mutants (P20−P279) were prepared using the NucleoBond Xtra Midi EF kit (Macherey Nagel, Germany). Transient transfection of the HEK293-6E cells was performed in triplicates at a cell density of 1.7 × 10^6^ cells mL^−1^ using a total of 1 μg plasmid DNA and 2 μg PEI MAX 40K (Polysciences, Inc., Germany) per mL culture volume. As negative control (mock), HEK293-6E cells were transfected using sterile H_2_O instead of plasmid DNA. Cells were fed with 0.5% (w/v) tryptone N1 (Organotechnie, France) and 5 mM valproic acid (Merck, Germany) 48 h post transfection. Culture supernatants were harvested by centrifugation (10000 g, 15 min) 120 h post-transfection. The supernatants were filtered (0.45 μM Supor membrane filter, Pall Corporation, U.S.A.) and diafiltrated against 20 mM phosphate buffer containing 500 mM NaCl and 20 mM imidazole (pH 7.4) using a Labscale TFF System equipped with a 30 kDa cutoff PelliconTM XL device (Merck, Germany). Subsequently, the diafiltrated sample was loaded onto a 1 mL HisTrap HP column (Cytiva, U.S.A.) and bound protein eluted applying a linear gradient of 20−500 mM imidazole over 20 column volumes using the ÄKTA pure chromatography system (Cytiva, U.S.A.). Pooled fractions containing the protein of interest were packed into a Spectra/Por Dialysis tubing (Spectrum Chemical Mfg. Corp., U.S.A.) and dialyzed against 20 mM phosphate buffer containing 200 mM NaCl (pH 7.4) at 4 °C overnight. The dialyzed protein was concentrated using Amicon Ultrafilters with a cutoff of 10K (Merck, Germany) and subjected to size exclusion chromatography (SEC) on a HiLoad 16/600 Superdex 200 pg column (Cytiva, U.S.A.) equilibrated with the same buffer used for dialysis. Purified proteins were stored at −80 °C until further use.

The single point mutation for the plasmid preparation of the R76T mutant was introduced using the QuikChange Lightning Site-Directed-Mutagenesis kit (Agilent Technologies, U.S.A.) according to the manufacturer’s instructions and the pTT5 vector containing the CD19-wt fusion construct as template. Recombinant expression and purification of the protein was performed as described above.

### Bio-Layer Interferometry (BLI) Measurements

Titers of the recombinant expressed CD19-wt and SF proteins performed in triplicates were determined using a biolayer interferometry assay on an Octet RED96e (ForteBio, U.S.A.). The entire experiment was performed at 25 °C using Anti-Penta-His biosensors (ForteBio, U.S.A.) with the plate shaking at 1000 rpm. Prior analysis, all solutions were filtered (0.45 μm) and the culture supernatants were centrifuged (16000 g, 10 min). The mock supernatant was spiked with 0.05% (v/v) Tween20 (mock solution, MS) and used for the baseline step and the sample dilutions and as negative control subtracted from each sample measurement. The biosensors were first equilibrated in neutralization solution (NS) containing PBS + 0.05% (v/v) Tween20 (pH 7.4), before they were dipped in MS for 600 s to record a baseline. For the subsequent association step, the biosensors were submerged in the culture supernatants diluted in MS for 150 s. The biosensors were regenerated in regeneration solution (RS) (10 mM glycine buffer, pH 1.5) and NS solution according to the manufacturer’s instructions. For each transfection a standard curve was prepared by spiking of purified SF05 in different concentrations (12−2.2 μg mL^−1^) in MS. Measurements were performed in triplicates. Analysis was performed using the Octet data analysis software version 11.1.1.39 (ForteBio, U.S.A.) according to the manufacturer’s guidelines.

### SDS-PAGE and Western Blot Analysis

SDS-PAGEs were either carried out using Bolt 4−12% Bis-Tris Plus Gels or 4−12% Bis-Tris Protein Gels and NuPAGE MOPS SDS Running Buffer (all from Thermo Fisher Scientific, U.S.A.). A total of 37 and 18 μL of the HEK293-6E supernatants of the respective transient transfection was used for the silver-stained SDS-PAGE and Western Blot analysis, respectively. For analysis of SEC purified proteins, a total of 500 ng of each SF variant was used. Prior loading onto the gel, each sample was mixed with NuPAGE LDS Sample buffer (4×; Thermo Fisher Scientific, U.S.A.) and heated at 70 °C for 10 min. After separation of the proteins by electrophoresis, bands were detected using a silver staining procedure as described previously.^60,61^ For Western Blot analysis, the proteins were electrotransferred onto a PVDF membrane (Carl Roth, Germany). Following a blocking step using milk powder (Carl Roth, Germany), the membrane was incubated with an anti 6x-His tag biotinylated monoclonal antibody (Thermo Fisher Scientific, U.S.A.), which was detected with a streptavidin-HRP conjugate (Cytiva, U.S.A.). For visualization of protein bands, the membrane was incubated with SuperSignal West Pico PLUS Chemiluminescent Substrate (Thermo Fisher Scientific, U.S.A.) according to the manufacturer’s protocol and subjected to the FUSION-FX7 spectra chemiluminescence imaging system (Vilber, France). As length standards, the PageRuler Unstained and Prestained Protein Ladders (both from Thermo Fisher Scientific, U.S.A.) were used as a size marker for SDS-PAGE and Western Blot analysis, respectively.

### Size-Exclusion Chromatography-Multiangle Light Scattering (SEC-MALS)

SEC-MALS was used to characterize the recombinant expressed SF mutants in solutions relating to their purity, native oligomers or aggregates and molar masses. Analyses were performed on an LC20 prominence HPLC system equipped with the refractive index detector RID-10A, the photodiode array detector SPD-M20A (all from Shimadzu, Japan), and a MALS Heleos Dawn8C plus QELS detector (Wyatt Technology, U.S.A.). A Superdex 200 10/300 GL column (Cytiva, U.S.A.) was used and equilibrated with PBS plus 200 mM NaCl (pH 7.4) as running buffer. Experiments were performed at a flow rate of 0.75 mL min^−1^ at 25 °C and analyzed using the ASTRA 6 software (Wyatt Technology, U.S.A.). Proper performance of the MALS was verified by the determination of the molar mass of a sample of bovine serum albumin. Prior to analysis, samples were thawed, centrifuged (16000 g, 10 min), and filtered by a 0.1 μm Ultrafree-MC filter (Merck Millipore, Germany). A total amount of 25 μg of the respective SF variant was injected for each measurement.

### Generation and Cultivation of CAR-T Cells

Buffy coats from anonymous healthy donor’s blood were purchased from the Austrian Red Cross, Vienna. CD3^+^ primary human T cells were isolated using the RosetteSep Human T cell Enrichment Cocktail (STEMCELL Technologies, Canada) and immediately cryopreserved in RPMI-1640 GlutaMAX medium (Thermo Fisher Scientific, U.S.A.) supplemented with 20% FCS and 10% DMSO (both from Merck, Germany). Primary human T cells were thawed in RPMI-1640 GlutaMAX medium, supplemented with 10% FCS, 1% penicillin-streptomycin (Thermo Fisher Scientific, USA) and 200 IU mL^−1^ recombinant human IL-2 (Peprotech, U.S.A.) and activated with Dynabeads Human T-Activator CD3/CD28 beads (Thermo Fisher Scientific, U.S.A.) at a 1:1 ratio according to the manufacturer’s instructions. A total of 24 h after stimulation, T cells were transduced in cell culture plates, which were coated with RetroNectin (Takara, Japan), according to the manufacturer’s instructions. Thawed lentiviral supernatant was added to the T cells at a final dilution of 1:2, yielding a cell concentration of 0.5 × 10^6^ cell mL^−1^. 48 h after transduction, selection of CAR-T cells was performed by treatment with 1 μg mL^−1^ puromycin (Merck, Germany) for 2 days. Transduced T cells were cultivated in AIMV medium (Thermo Fisher Scientific, U.S.A.) supplemented with 2% Octaplas (Octapharma, Switzerland), 1% l-glutamine, 2.5% HEPES (both from Thermo Fisher Scientific, U.S.A.) and 200 IU mL^−1^ recombinant human IL-2.

### Construction of Chimeric Antigen Receptor (CAR) Genes

The FMC63.4−1BB.ζ CAR was constructed by linking the sequences from the signal peptide derived from the human granulocyte-macrophage colony-stimulating factor receptor subunit α (GM-CSF-R-α; Uniprot P15509, aa 1−22) to the CD19-specific scFv derived from the FMC63 antibody. This sequence was synthesized (GenScript, U.S.A.) and subcloned into an pCDH-based lentiviral backbone plasmid (System Biosciences, U.S.A.) encoding the stalk and transmembrane domain of CD8α (Uniprot P01732, aa 138−206), the intracellular domains of 4−1BB (UniProt Q07011 aa 214−255) and CD3ζ (Uniprot P20963−3, aa 52−164, additional mutation Q65K). The resulting CAR was linked to the puromycin N-acetyl transferase for efficient selection of CAR^pos^ T cells and the fluorescence protein iRFP713 (for verification of transduction and expression) by ribosomal skipping sequences (T2A and P2A, respectively).^62^

### Construction of Lentiviral Vector

VSV-G pseudotyped lentivirus was generated by cotransfection of Lenti-X 293T cells (Takara, Japan) with a puromycin-selectable pCDH expression vector (System Biosciences, U.S.A.) encoding the second-generation anti CD19-CAR (FMC63.4−1BB.ζ) and viral packaging plasmids pMD2.G and psPAX2 (Addgene plasmids #12259 and #12260, respectively; gifts from Didier Trono) using the PureFection Transfection Reagent (System Biosciences, U.S.A.) according to the manufacturer’s instructions. Viral supernatants were collected on days 2 and 3 after transfection and were concentrated 100-fold using the Lenti-X Concentrator (Takara, Japan) according to the manufacturer’s instructions. Viral suspensions were frozen at −80 °C until further use.

### Flow Cytometric Analysis of Binding of SF05 to CD19-Directed CAR-T Cells

A total of 1 × 10^5^ CAR-T cells was taken for flow cytometric analysis. Cells were washed once in FACS buffer composed of PBS supplemented with 0.2% human albumin (CSL Behring, U.S.A.) and 0.02% sodium azide (Merck, Germany). Subsequently, the cells were incubated with different concentrations (274/91/30/10/3/1 and 0 nM) of AF647-labeled (Thermo Fisher Scientific, U.S.A.) and SEC-purified SF05 (SF05-AF647) for 25 min at 4 °C. Cells were washed twice with ice-cold FACS buffer and data were acquired on an LSR Fortessa instrument (BD Biosciences, U.S.A.) and analyzed using the FlowJo software (BD Biosciences, U.S.A.). Donor-matched nontransduced cells were likewise incubated with SF05-AF647 in the same concentration range (274−0 nM) and used as a negative control. The fluorescence protein iRFP713, which was also expressed by the CAR-T cells, emitted fluorescence light in the same channel that was also used for SF05-detection. However, the iRFP713 signal of CAR-T cells in the absence of SF05 was almost negligible compared with that obtained with SF05 (∼2% of the signal obtained with 91 nM SF05; Figure 5A). Nevertheless, to exclude also this minor interference, the signal of non-SF05-labeled CAR-T cells was subtracted from all data points for determination of the *K*_D_ value. T cells from three different donors were analyzed in two independent experiments.

Immunophenotyping of the peripheral blood (PB) of CAR-T cell recipients suffering from diffuse large B cell lymphoma and receiving tisagenlecleucel (Kymriah) was performed according to standard procedures. Briefly, erythrocytes were lysed by the NH_4_Cl method and 1.5 × 10^6^ leukocytes in 50 μL of flow cytometry buffer (PBS, 0.5% BSA and 0.05% NaN_3_) were incubated with 7 μg mL^−1^ biotinylated and SEC-purified SF05 (SF05-biotin) at 4 °C for 25 min. For control purposes, cells were incubated with CD19 CAR detection reagent (Miltenyi, Bergisch Gladbach) or flow cytometry buffer alone. Subsequently, leukocytes were washed twice and incubated with the combination of directly conjugated monoclonal antibodies CD3-PECy7 (UCHT1), CD4-PE-eFluor610 (RPA-T4), CD14-PerCP-eFluor710 (61D3), CD38-APC-eFluor780 (HIT2), CD45-eFluor506 (HI30), and HLA-DR-eFluor450 (LN3; all obtained from Thermo Fisher Scientific, U.S.A.) and CD15-FITC (W6D3, Biolegend, U.S.A.) and streptavidin PE (Thermo Fisher Scientific, U.S.A.), to visualize the binding of the biotin-conjugated SF05 and the CD19 CAR detection reagent at 4 °C for 25 min. Subsequently, cells were washed twice and acquisition was performed on a fluorescence activated cell sorter (Navios, Beckman Coulter, Krefeld, Germany) supported by the Kaluza software. Acquired data were analyzed with the FlowJo software (BD Biosciences, U.S.A.). Analysis of patient data was approved by the EC of the Medical University of Vienna (EC No.: 1290/2020).

## Supporting Information Available

Effect of thermal denaturation on binding of anti-HA mAb, anti-c-myc mAb, FMC63, and HIB19 (Figure S1); characterization of libraries M and XL (Figure S2); analysis of single SF mutants (Figure S3) (PDF)
