# Supplementary material for: Directed Evolution of Stabilized Monomeric CD19 for Monovalent CAR Interaction Studies and Monitoring of CAR-T Cell Patients
Source: ACS Synth Biol. 2021 Apr 12;10(5):1184–98. doi: 10.1021/acssynbio.1c00010 (PMC8155657; doi:10.1021/acssynbio.1c00010)
Supplement: Supplementary file 1 — sb1c00010_si_001.pdf [file sb1c00010_si_001.pdf]

**SUPPORTING INFORMATION**

# Directed evolution of stabilized monomeric CD19 for monovalent CAR interaction studies and monitoring of CAR-T cell patients

Elisabeth Laurent, Anna Sieber, Benjamin Salzer, Anna Wachernig, Jacqueline Seigner, Manfred Lehner, René Geyeregger, Bernhard Kratzer, Ulrich Jäger, Renate Kunert, Winfried F. Pickl, Michael W. Traxlmayr*

*Corresponding Author:
Department of Chemistry, BOKU - University of Natural Resources and Life Sciences, Muthgasse 18, 1190 Vienna, Austria; orcid.org/0000-0002-2108-582X; Phone: +43 1 47654 77274; Email: michael.traxlmayr@boku.ac.at

**CONTENTS**

Figure S1. Effect of thermal denaturation on binding of different mAbs.
Figure S2. Characterization of libraries M and XL by sequencing of 48 clones, respectively.
Figure S3. One hot spot mutation is sufficient to improve the structural integrity and thermal stability of CD19-ECD.

## SUPPLEMENTARY FIGURES

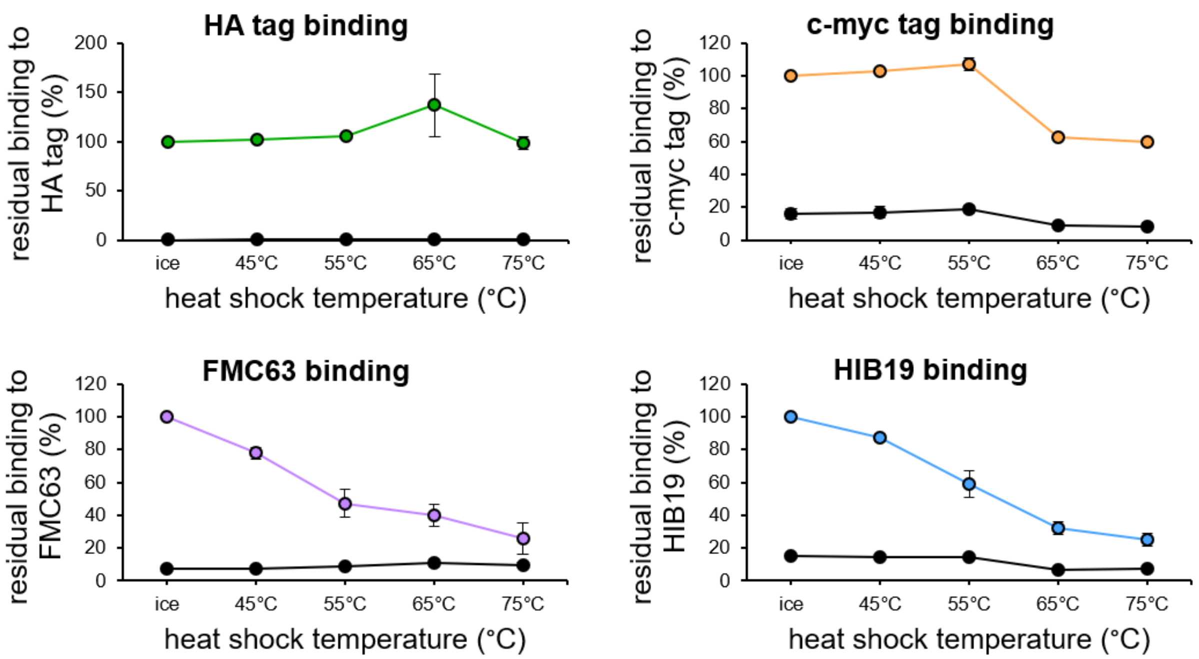


**Figure S1. Effect of thermal denaturation on binding of different mAbs.** Flow cytometric analysis of yeast cells displaying CD19-wt after incubation at increasing temperatures for 10 min and staining with anti-HA mAb, anti-c-myc mAb, FMC63 and HIB19, respectively. The black curve represents the negative control [staining with secondary antibody only (for FMC63 and anti-c-myc) or cellular autofluorescence (for anti-HA and HIB19, which were both directly labeled]**.** The residual binding of the mAbs is plotted versus the incubation temperature (normalized to the mean of non-heated yeast cells binding to the respective mAb). Averages ± SDs of three independent experiments are shown.

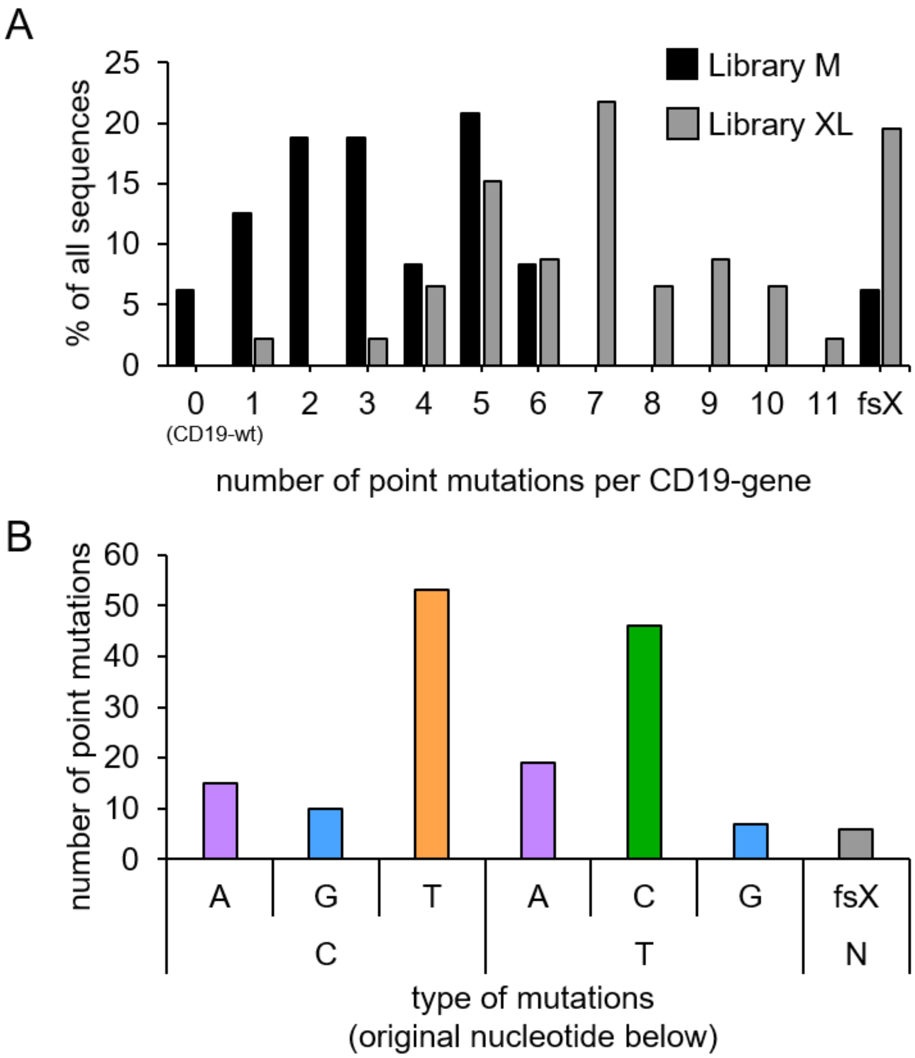


**Figure S2. Characterization of libraries M and XL by sequencing of 48 clones, respectively. (A)** The frequency of detected nucleotide mutations over the CD19-wt gene (P20-P278) for both libraries is shown. **(B)** The number of each possible nucleotide mutation found in library M is shown. fsX refers to frameshift mutations.

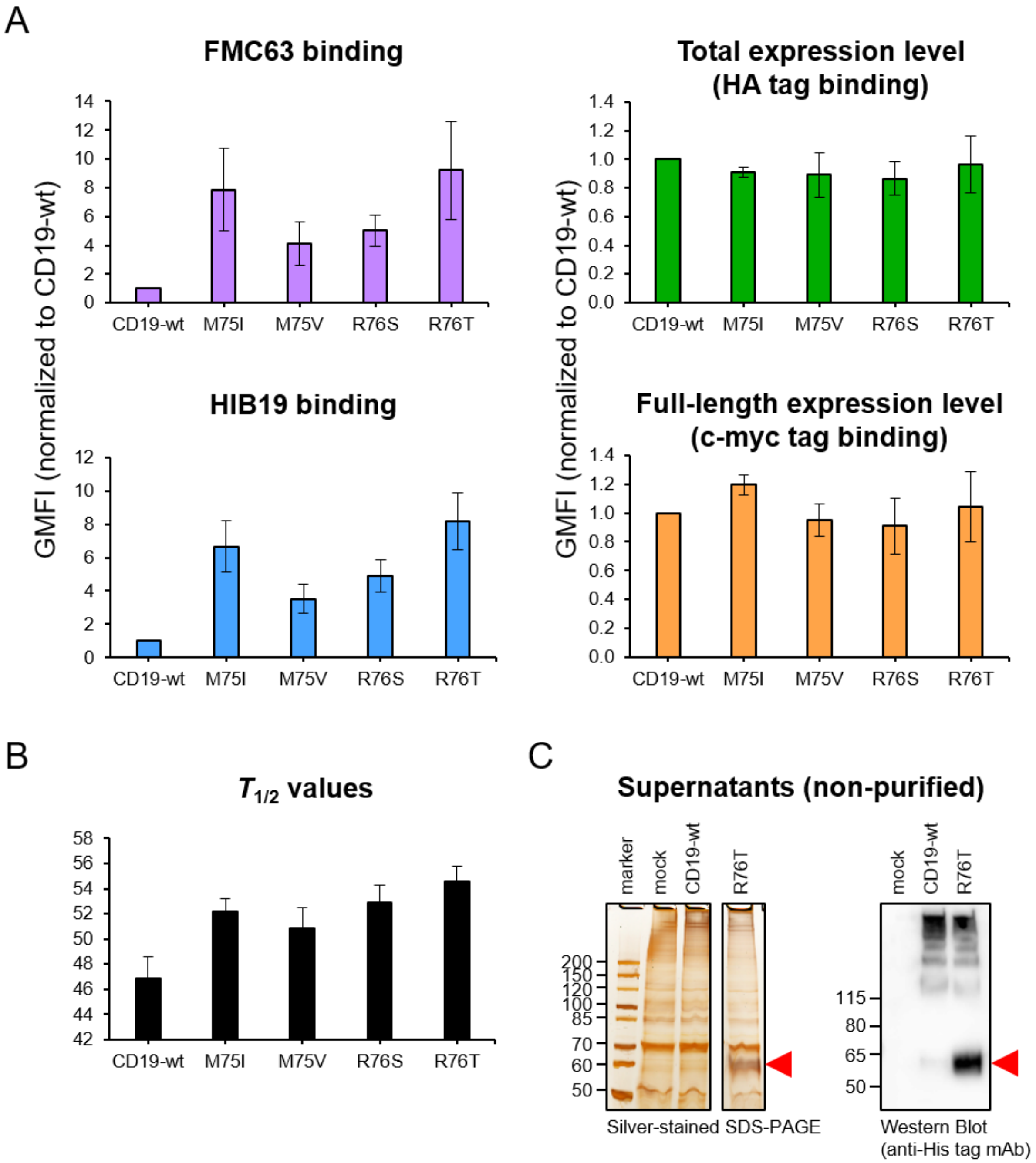


**Figure S3. One hot spot mutation is sufficient to improve the structural integrity and thermal stability of CD19-ECD. (A)** Analysis of binding properties of single SF mutants (M75I, M75V, R76S, R76T) relative to CD19-wt. Displaying yeast cells were probed for binding to the structure specific anti-CD19-ECD mAbs (FMC63 and HIB19) and the anti-tag mAbs (anti-HA and anti-c-myc) by flow cytometry. **(B)** Determination of the $T_{1/2}$ values of single SF mutants relative to

CD19-wt. Displaying yeast cells were incubated at increasing temperatures for 10 min and subsequently analyzed for their binding to HIB19 by flow cytometry. Resulting data allow the calculation of $T_{1/2}$ values based on modelled denaturation curves.[1,2] The plot shows calculated $T_{1/2}$ values of CD19-wt and single SF variants. All data in this figure represent averages ± SDs of three independent experiments. **(C)** Analysis of the supernatant of HEK293-6E cells transiently transfected with plasmids encoding CD19-wt or the R76T single SF mutant by silver-stained SDS-PAGE and Western Blot detecting His-tagged protein. Mock (negative control) refers to cells that were transfected with sterile $H_2O$ instead of plasmid DNA. The red marks indicate migration of the monomeric CD19-ECD protein.

## REFERENCES

[1] Orr, B. A., Carr, L. M., Wittrup, K. D., Roy, E. J., and Kranz, D. M. (2003) Rapid method for measuring ScFv thermal stability by yeast surface display, *Biotechnology progress 19*, 631-638.

[2] Traxlmayr, M. W., and Shusta, E. V. (2017) Directed Evolution of Protein Thermal Stability Using Yeast Surface Display, *Methods Mol Biol 1575*, 45-65.
